# Comparison of intravaginal progesterone gel and intramuscular 17-α-hydroxyprogesterone caproate in luteal phase support

**DOI:** 10.3892/etm.2013.1049

**Published:** 2013-04-04

**Authors:** FUNDA SATIR, TAYFUN TOPTAS, MURAT INEL, MUNIRE ERMAN-AKAR, OMUR TASKIN

**Affiliations:** Department of Obstetrics and Gynaecology, Akdeniz University Hospital, Antalya 07070, Turkey

**Keywords:** intravaginal progesterone gel, 17-α-hydroxyprogesterone, luteal phase support

## Abstract

The main objective of this study was to compare the pregnancy rates of intramuscular (IM) 17-α-hydroxyprogesterone caproate (17-HPC) and intravaginal (IV) progesterone gel administration in *in vitro* fertilization-embryo transfer (IVF-ET) cycles. The IM 17-HPC and IV progesterone groups included 632 (66.4%) and 320 (33.6%) women undergoing the first cycles of IVF-ET treatment, respectively. Multivariate analyses annotated for all potential confounders showed that the use of IV progesterone retained a predictive value for the total β-human chorionic gonadotropin (hCG) positivity and clinical pregnancy rates [adjusted odds ratio (OR), 1.97; 95% confidence interval (CI), 1.28–3.03; P=0.002; and OR, 1.66; 95% CI, 1.07–2.60; P=0.03, respectively]. However, biochemical and on-going pregnancy rates did not differ significantly between the groups (OR, 1.85; 95% CI, 1.00–3.41; P=0.05; and OR, 1.43, 95% CI, 0.89–2.30; P=0.14, respectively). Luteal phase support (LPS) with IV progesterone gel in comparison with IM 17-HPC appears to be associated with higher clinical pregnancy rates in IVF-ET cycles. However, this benefit is clinically irrelevant in terms of on-going pregnancy outcomes.

## Introduction

Ovarian hyperstimulation procedures during *in vitro* fertilization-embryo transfer (IVF-ET) cycles stimulate the formation of multiple follicles and corpora lutea. However, the quantity of endogenous progesterone produced by multiple corpora lutea is not sufficient for the implantation and the continuation of early pregnancy (1–3). There are several mechanisms leading to luteal phase defect in IVF-ET cycles. The use of gonadotropin-releasing hormone (GnRH) analogs in IVF-ET cycles suppresses the production of endogenous gonadotropins. This, in particular due to the suppression of luteinizing hormone (LH), halts the activity of the corpus luteum (4). Furthermore, GnRH antagonists may also shorten the luteal phase. Human chorionic gonadotropin (hCG) has a negative feedback on the pituitary gland, and causes pituitary suppression during the luteal phase (5). Thus, it is necessary to use exogenous progesterone to support the luteal phase in IVF-ET cycles due to the insufficient corpus luteum function (1–3,6). In addition, the American Society for Reproductive Medicine (ASRM) Position Statement recommends progesterone supplementation in IVF cycles due to the higher pregnancy rates achieved by progesterone administration compared with placebo or no treatment (1).

Among the routes of progesterone administration, intravaginal (IV) and intramuscular (IM) routes are currently the most commonly used (7,8). Although IM forms of progesterone achieve higher serum progesterone levels, IV administration provides higher local tissue levels in the endometrium (9). A previously published meta-analysis demonstrated the benefit of IM over IV progesterone administration for luteal phase support (LPS) on pregnancy outcomes (6). However, another meta-analysis including additional randomized controlled studies showed that the effects of IV and IM forms of progesterone on pregnancy end-points were comparable (10). Notably, the most effective route of progesterone administration in LPS remains to be clarified.

The aim of the current study was to compare the effects of IM and IV gel forms of progesterone used for LPS following IVF-ET on clinical and on-going pregnancy rates.

## Patients and methods

### Patients

This retrospective single centre study was undertaken in subjects who received IVF-ET treatment between October 1999 to August 2009 in the Department of Obstetrics and Gynaecology, Centre for Assisted Reproductive Techniques and Infertility, Akdeniz University Hospital, Antalya, Turkey. Informed consent was obtained from all patients. Medical charts of all patients were reviewed, and data concerning the medical and infertility-related history, pelvic examination, treatment modalities and follow-up were recorded. The ethics committee of Akdeniz University Hospital approved the study according to the Good Clinical Practice guidelines of the International Conference on Harmonization and national regulations (11).

### Procedures

Controlled ovarian hyperstimulation (COH) was applied according to the long protocol-GnRH analog or antagonist protocol (12). Oocyte pick-up (OPU) was performed 36 h after hCG injection. Starting from the night of the OPU, patients received IV or IM progesterone and slow oscillating transdermal oestrogen (Climara^®^ Forte 7.8 mg; Schering German, Istanbul, Turkey) for LPS, until the 12th day after ET when pregnancy tests were performed. LPS was provided by the administration of 90 mg/day IV progesterone (Crinone^®^ 8% vaginal gel; Serono, Istanbul, Turkey) or 250 mg IM 17-α-hydroxyprogesterone caproate (17-HPC; Proluton depot^®^ ampules; Bayer Schering, Istanbul, Turkey) every 3 days. Serum β-hCG was analyzed on the 12th day after ET. Endometrial thickness was measured on the day of hCG administration.

### Study outcomes

The primary outcome of the study was on-going pregnancy rate. Secondary endpoints included β-hCG positivity, biochemical pregnancy and clinical pregnancy rates. Biochemical pregnancy was defined as the absence of clinical and sonographical evidence of pregnancy despite positive β-hCG values (>10 mIU/ml). Clinical pregnancies were exclusive of biochemical and ectopic pregnancies and required a detectable intrauterine gestational sac on ultrasound examination. On-going pregnancies were defined by the presence of intrauterine embryonic heart activity, as determined by transvaginal ultrasonography.

### Statistical methods

Demographic and clinical characteristics are reported with descriptive analysis. Normality of data distribution was assessed by the Shapiro-Wilk test. All continuous variables were dichotomized by means of median values. Mann-Whitney U test, Student’s t-test, and Chi-square tests were used for univariate comparisons. Variables with a P<0.05 in univariate analysis were included in the logistic regression analysis. A P-value of <0.05 was required to reject the null hypothesis. Effects on pregnancy rates were reported by adjusted odds ratios (ORs) and 95% confidence intervals (CIs). Post hoc power analysis revealed an OR of 0.5 with a power of 100% and an overall two-sided type I error of 5%, with inclusion of a total of 952 women in a two-sided Wald test. All data management and analyses were performed using Stata version 11 (Stata Corp., College Station, TX, USA) and Prism version 5.0 for Mac OSX (GraphPad software Inc., La Jolla, CA, USA).

## Results

A total of 952 women in their first IVF-ET cycles were included in the study. The IM 17-HPC group consisted of 632 women (66.4%) and the other 320 (33.6%) patients received IV Crinone 8% gel. A total of 25 cycles were cancelled: 15 (2.4%) cycles in 17-HPC group and 10 (3.1%) cycles in the Crinone group (P=0.49). Thus, final analyses were performed in a total of 927 patients, including 617 patients in the 17-HPC group and 310 in the Crinone group. The main clinical and demographic data of the patients are shown in Table I.

The median age was 32 years (range, 20–46 years). The 17-HPC group was older (32 vs. 31 years of age, P=0.003); had higher basal FSH (6.6 vs. 6.4 IU/l, P=0.01), basal estradiol (E2) [305 pmol/l (83 pg/ml) vs. 283 pmol/l (77 pg/ml), P<0.001], and serum E2 levels on the day of hCG administration [13,928 pmol/l (3,794 pg/ml) vs. 12,817 pmol/l (3,492 pg/ml), P<0.001]; had a longer duration of gonadotropin stimulation (12.24 vs. 11.90 days, P<0.001), higher total dose of gonadotropins (3,700 vs. 3,413 IU, P<0.001) and thicker endometrial thickness (8.3 vs. 7.6 mm, P<.001). The median numbers of retrieved oocytes and transferred embryos were also higher in the 17-HPC group (P=0.03 and P<0.001, respectively). The groups were comparable for the remaining demographic characteristics including infertility periods, infertility etiology and IVF indications. The most frequent IVF-ET indication in the two groups was ovulatory dysfunction (Table I).

**Figure 1:**
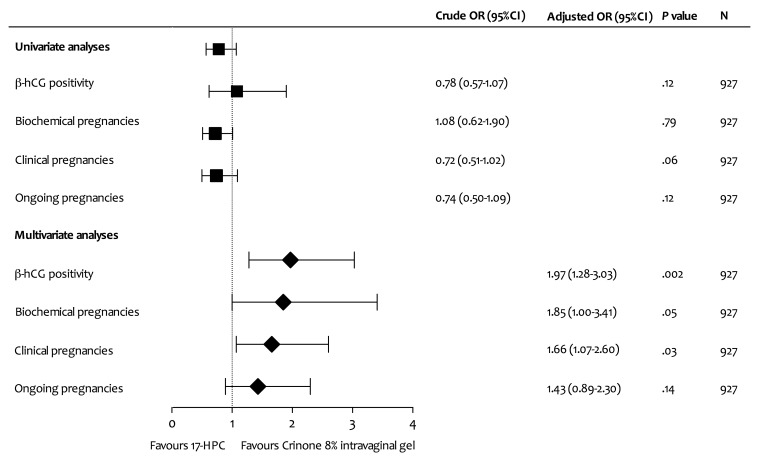
Univariate and multivariate analyses of dataset. OR, odds ratio; CI, confidence interval; 17-HPC, 17-α-hydroxyprogesterone caproate; hCG, human chorionic gonadotropin.

There were no statistically significant differences between the Crinone and 17-HPC groups with respect to total β-hCG positivity, biochemical pregnancy, clinical pregnancy and on-going pregnancy rates in univariate analyses (Fig. 1). In multivariate regression analysis, the use of Crinone was associated with higher β-hCG positivity and clinical pregnancy rates (OR, 1.97; 95% CI, 1.28–3.03; P=0.002; and OR, 1.66; 95% CI, 1.07–2.60; P=0.03, respectively). There was a trend toward higher biochemical pregnancy rates favouring Crinone 8% IV gel (OR, 1.85; 95% CI, 1.00–3.41; P=0.05). However, no statistically significant difference was found in terms of on-going pregnancy rates between the groups (OR, 1.43; 95% CI, 0.89–2.30; P=0.14; Fig. 1).

## Discussion

This analysis shows that Crinone vaginal gel is associated with comparable pregnancy outcomes to IM progesterone, 17-HPC. To the best of our knowledge, this is the largest study comparing these two forms of luteal phase progesterone support in IVF.

Progesterones may be administered via the oral, vaginal, or IM routes for LPS following IVF-ET. Despite it being an easy-to-use form, oral progesterone support provides lower implantation rates than other forms of progesterone (13,14). It has unpleasant side-effects, including nausea, fluid retention, sedation, drowsiness and other hypnotic effects as a result of metabolites generated by the first-pass effect (15,16). At present, oral progesterone is not a preferred method for LPS following IVF-ET. IM progesterone in oil has higher rates of clinical and on-going pregnancies compared with placebo or oral forms of progesterone (17–19). However, the IM progesterone-in-oil form has also various side-effects, including serious inflammatory reactions, sterile abscesses and significant patient discomfort. These side-effects may last for long periods of time, even after the discontinuation of the drug (20).

17-HPC, a slow-release, long-acting derivative of progesterone, may hypothetically be regarded as a valid alternative to the IM progesterone-in-oil form with an advantage of reduced patient discomfort. It has been found to be comparable to the IM progesterone-in-oil form with respect to clinical or on-going pregnancy rates in a prospective randomized study (20). However, evidence regarding the equivalent efficacy between the two formulations is limited.

The most commonly used vaginal regimens include 600 mg progesterone (200 mg three times a day) as an oil-in-capsule formulation and daily 90 mg progesterone in a polycarbophil-containing bioadhesive gel form (Crinone 8%). These formulations have similar pregnancy outcomes and minor side-effects in LPS (21–23).

Currently, standard progesterone administration during LPS is provided by either the vaginal (as an oil-in-capsule or bioadhesive gel form) or IM route (50 mg a day). Previously, a number of studies sought to evaluate the optimal route of progesterone administration during LPS (24–27). Based on a meta-analysis of nine prospective randomized controlled trials that included a total of 1,620 individuals, vaginal and IM in-oil forms of progesterone applied during LPS are comparable in terms of clinical pregnancy rate (34.2 vs. 36.3%; OR, 0.91; 95% CI, 0.74–1.13) and on-going pregnancy rate (25.3 vs. 26.5%; OR, 0.94; 95% CI, 0.71–1.26) (12). Overall, this meta-analysis and most of the prospective trials included support the results of univariate analysis in the current study. The only prospective study comparing 17-HPC with Crinone demonstrated superior efficacy with 17-HPC (28). Discrepancies between the results of our study and previous studies may be attributed to the method of dealing with confounding factors. Differences in randomization strategies used in prospective studies and multivariate regression modelling, if any, in retrospective analyses are the main cause of heterogeneity in the results of various studies.

We conclude that LPS with IV progesterone gel in comparison with IM 17-HPC appears to be associated with better clinical pregnancy rates in IVF-ET cycles. However, this benefit is clinically irrelevant in terms of on-going pregnancy outcomes.

## Figures and Tables

**Table I:** Characteristics of IVF groups.

Variables	IM 17-HPC (n=632)	Crinone 8% gel (n=320)	P-value
Age, years			0.003
Median	32	31	
Interquartile range	29–36	28–35	
Duration of infertility, years			0.14
Median	7	6	
Interquartile range	5–9	4–9	
Etiology of infertility, n (%)			0.56
Primary	561 (88.8)	288 (90.0)	
Secondary	71 (11.2)	32 (10.0)	
IVF indications, n (%)			0.06
Male factor	81 (12.8)	35 (10.9)	
Tubal factor	102 (16.1)	46 (14.4)	
Ovulatory dysfunction	216 (34.2)	137 (42.8)	
Endometriosis	76 (12.0)	31 (9.7)	
Male and female factor	13 (2.1)	14 (4.4)	
Advanced maternal age	30 (4.8)	10 (3.1)	
Unexplained	93 (14.7)	41 (12.8)	
Other	21 (3.3)	6 (1.9)	
Cancelled cycles, n (%)	15 (2.4)	10 (3.1)	0.49
Basal FSH, IU/l			0.01
Median	6.6	6.4	
Interquartile range	5.4–8.0	4.2–7.8	
Basal E2, pmol/l (pg/ml)			<0.001
Median	305 (83.0)	283 (77.0)	
Interquartile range	268–330 (73.0–90.0)	202–340 (55.0–93.0)	
Duration of gonadotropin stimulation, days			<0.001
Median	12.24	11.90	
Standard deviation	1.08	1.45	
Number of retrieved oocytes			0.03
Median	18	17	
Interquartile range	15–21	13–21	
Number of transferred embryos			<0.001
Median	3	3	
Interquartile range	3–3	2–3	
E2 levels^a^, pmol/l (pg/ml)			<0.001
Median	13928 (3794)	12817 (3492)	
Interquartile range	11237–16988 (3061–4628)	10336–15695 (2816–4276)	
Total dose of gonadotropin, IU			<0.001
Median	3700	3413	
Interquartile range	3000–4375	2750–4075	
Endometrial thickness, mm			<0.001
Median	8.3	7.6	
Interquartile range	7.6–9.4	7.1–8.4	

IVF, *in vitro* fertilization; FSH, follicle-stimulating hormone; E2, estradiol; IM, intramuscular; 17-HPC, 17-α-hydroxyprogesterone caproate; hCG, human chorionic gonadotropin.

a E2 levels on the day of hCG administration.
